# Supplementary material for: Degenerative Cervical Myelopathy: Insights into Its Pathobiology and Molecular Mechanisms
Source: J Clin Med. 2021 Mar 15;10(6):1214. doi: 10.3390/jcm10061214 (PMC8001572; doi:10.3390/jcm10061214)
Supplement: Supplementary file 1 [file jcm-10-01214-s001.pdf]

Supplementary file

Table S1. Normal range of motion of the cervical spine. One of the first findings in DCM conditions is a limited range of motion, often due to pain and this finding can appear even before any radiologic changes. The decrease in range of motion is often subtle at first and a significant limitation is usually present before the patient seeks medical attention for this cause [14].

Movement	Average (normal) range of motion	Main joint(s) involved
Flexion	90°	C0-C1 (10 °), Sub-axial cervical spine (60°)
Extension	70°	C0-C1 (21°), Sub-axial cervical spine (40°)
Lateral Bending	20-45°	Sub-axial cervical spine (10-35°)
Axial Rotation	90° each side	C1-C2 (40°)
